# Thin Film Deposition Using Energetic Ions

**DOI:** 10.3390/ma3084109

**Published:** 2010-07-29

**Authors:** Darina Manova, Jürgen W. Gerlach, Stephan Mändl

**Affiliations:** Leibniz-Institut für Oberflächenmodifizierung, Permoserstr. 15, 04318 Leipzig, Germany; E-Mails: darina.manova@iom-leipzig.de (D.M.); juergen.gerlach@iom-leipzig.de (J.W.G.)

**Keywords:** PVD, thin films, ion assisted deposition, ion implantation, nanostructures

## Abstract

One important recent trend in deposition technology is the continuous expansion of available processes towards higher ion assistance with the subsequent beneficial effects to film properties. Nowadays, a multitude of processes, including laser ablation and deposition, vacuum arc deposition, ion assisted deposition, high power impulse magnetron sputtering and plasma immersion ion implantation, are available. However, there are obstacles to overcome in all technologies, including line-of-sight processes, particle contaminations and low growth rates, which lead to ongoing process refinements and development of new methods. Concerning the deposited thin films, control of energetic ion bombardment leads to improved adhesion, reduced substrate temperatures, control of intrinsic stress within the films as well as adjustment of surface texture, phase formation and nanotopography. This review illustrates recent trends for both areas; plasma process and solid state surface processes.

## 1. Introduction

In the last decades, thin film deposition evolved into a ubiquitously present technology [1,2], penetrating all major industries. Besides semiconductors [3,4], optoelectronics [5,6], optics [7,8] and aerospace [9,10], the whole machine tool industry [11,12] depends on coatings. Correspondingly, a large variety of thin film deposition methods is available, ranging from paints and varnishes often employed for corrosion protection [13,14], dip or spin-coating commonly used for sol-gel processes [15,16], anodic oxidation for decorative consumer goods [17,18], for different vacuum processes [19,20]. By avoiding the presence of a background gas at low and very low pressures, both incorporation of impurities and scattering processes of particles are avoided, or at least minimized, in the latter group. Consequently, kinetic energies beyond the thermodynamic equilibrium are realized across several orders of magnitude.

While a large amount of literature is available describing the different processes and methods, with a different set of literature detailing the film properties and applications, a short and concise overview of processes and feasible influences on film properties, including recent advances in nanotechnology, can seldom be found. Current overviews could be consulted on more specific details of selected aspects, e.g., for the microstructural evolution during film growth detailing the influence of temperature, reactive species and ion irradiation [21,22], for ionized physical vapor deposition [23] or stress generation and relief processes [24]. In this manuscript, an attempt is made to give an introduction into different deposition technologies involving energetic ions with kinetic energies in the range from 10 to 10,000 eV, corresponding to temperatures of tens of thousands of Kelvins for the lower range (in SI units: 1 eV ≡ 1.602 × 10^−19^ J = 11,604.505 k_B_T, *k_B_* being the Boltzmann constant).

Non-equilibrium processes are expected to dominate in the ion-surface interactions encountered during these energetic deposition processes, including ion mixing, surface sputtering and the formation of metastable phases, effects which all can be employed in modern functional coatings [25]. The understanding of the phenomenology and mechanisms is necessary to develop new structures and applications in a fast and efficient way, in contrast to the empirical exploration of a vast parameter space.

However, before presenting the underlying mechanisms and a highly selective assortment of applications currently in development in various laboratories around the world, a short excurse into fundamentals of deposition processes employing energetic ions is necessary. There, different possibilities to achieve fast and shape independent coating of large areas, together with their respective pitfalls are presented in addition to a short raison d’être why energetic ions are employed at all.

## 2. Physical Vapor Deposition

**Figure 1:**
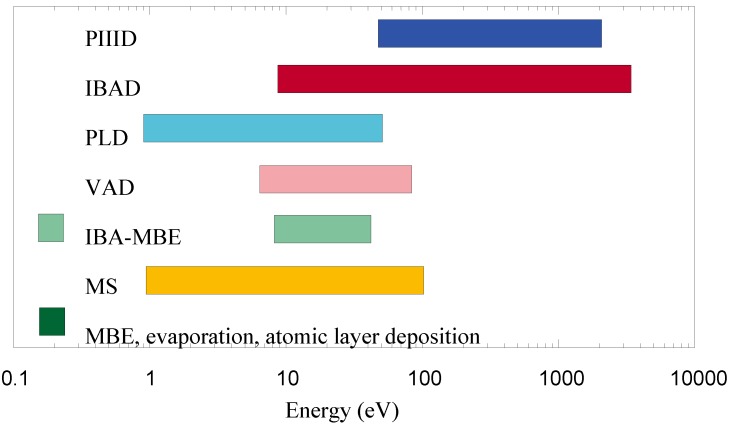
Typical energy ranges for different PVD processes. PIIID = plasma immersion ion implantation and deposition; IBAD = ion beam assisted deposition; PLD = pulsed laser deposition; VAD = vacuum arc deposition; IBA-MBE = ion beam assisted molecular beam epitaxy; MS = magnetron sputtering; MBE = molecular beam epitaxy.

Physical vapor deposition (PVD) is an established technology to obtain individually tailored surface coatings on various substrates [2,26]. For growth of epitaxial semiconductors, molecular beam epitaxy (MBE) or hyperthermal ion beam assisted MBE (IBA-MBE) is the method of choice [27,28], while optical coatings are mainly produced by magnetron sputtering [6,17]. Hard and wear resistant coatings with very high deposition rates are obtained from either magnetron sputtering (MS) or vacuum arc deposition (VAD) [29,30]. For pulsed laser deposition (PLD), similar ion energies compared to arc process are encountered [31,32], while ion beam assisted deposition (IBAD) [33,34] or plasma immersion ion implantation and deposition (PIIID) [35,36] leads to even higher average particle energies (see Figure 1 for a schematic overview of the typical energy ranges).

**Figure 2:**
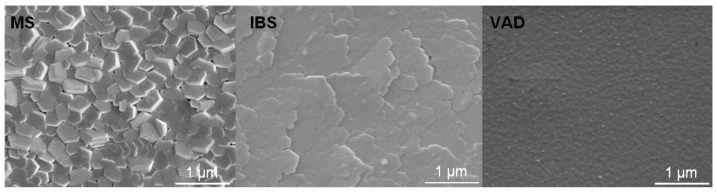
Morphology of pure Mg films deposited by magnetron sputtering (MS; left panel), ion beam sputtering (IBS; middle panel) and cathodic (vacuum) arc deposition (VAD; right panel) [37].

One of the most important parameters is the average energy per incoming particle where higher values lead to an increased surface mobility [33,38]. With increasing the deposited energy per particle, a higher (transient) mobility of the atoms impinging on the surface is obtained, thus increasing the apparent surface temperature [39]. At identical deposition rates, the larger mobility will lead to a higher diffusivity, thus allowing a further transport from the original arrival site and earlier coalescence [40]. Hence, changes in the surface morphology and texture are observed with higher energies leading to films with larger grains and less defects while the momentum of the incoming particles can lead to alignment or orientation of the growing crystallites [41,42], as shown in Figure 2.

**Figure 3:**
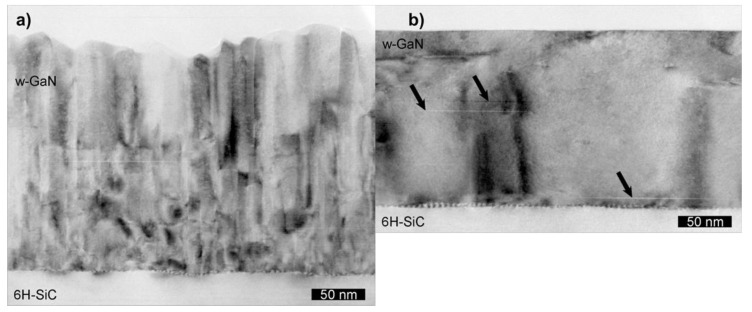
Cross section transmission electron microscopy (TEM) bright field images of (a) an MBE (without) and (b) an IBA-MBE (with additional ion assistance) grown GaN (0001) thin film on 6H-SiC (0001) [48].

With increasing average ion energy, a transition from columnar growth to layer-by-layer growth is observed [43,44], e.g., for GaN on SiC depicted in Figure 3. When comparing the incident ion energy with the displacement energy of surface and bulk atoms, the ion bombardment can be tailored to enhance the surface mobility during growth without leading to defect generation in the bulk below the surface: the transferred energy has to be between the surface threshold and the bulk threshold for displacements [45]. Additionally, this non-thermal energy can be used to reduce the substrate temperature during film growth due to the supplementary surface mobility [46]. In order to establish enhanced surface mobility without creation of bulk defects, a maximum ion energy—not average particle energy—of 10–80 eV is acceptable depending on the materials system, with several deposition methods allowing such an energy [44]. At the same time, a high degree of ionization is envisaged to permit a more direct control of particle momentum, direction, and magnitude [47].

### 2.1. High Power Impulse Magnetron Sputtering

Magnetron sputtering uses magnetic fields to enhance and confine the plasma close to the source of the material to be deposited, *i.e.*, target or cathode. Ion bombardment leads to the vaporization of these species with typical energies of a few eV, while only a small fraction of about 1% of the particles are actually ionized. Magnetron sputtering [49], with direct current or rf current, is a widely used technique for deposition of metal and compound layers, with the maximum power determined by the thermal load on the target, provided by the bombardment with positively charged ions. Typical operating parameters are a working pressure of 0.1–1 Pa, magnetic field strength of 0.01–0.05 T, cathode potentials of 300–700 V, yielding electron densities near the substrate of 10^15^–10^16^ m^-3^ [23]. Furthermore, a low fraction of around 1% of the sputtered material is ionized and the majority are the ions of the inert (or reactive) gas used for sputtering [6].

As a first step, decreasing the duty cycle allows a corresponding increase in power during the on-time. When a power density about two orders of magnitude higher than for conventional sputtering is maintained, a qualitative new process, termed high power impulse magnetron sputtering (HIPIMS) is observed [50,51]. Here, typical pulse lengths of 10–400 µs are used with pulse frequencies in the range of 50–500 Hz, yield a duty cycle around 0.5–5% at instantaneous power densities larger than 1 kW/cm^2^ [52,53]. At these greatly enhanced power densities, ionization of sputtered atoms occurs much more frequently than in conventional magnetron sputtering, thus increasing the fraction of ionized sputter material and reducing the necessary amount of sputter gas [54]. In the extreme case, gasless sputtering, using for instance a pulsed cathodic arc for initiation of the self-sputter mode, is achieved [55].

**Figure 4:**
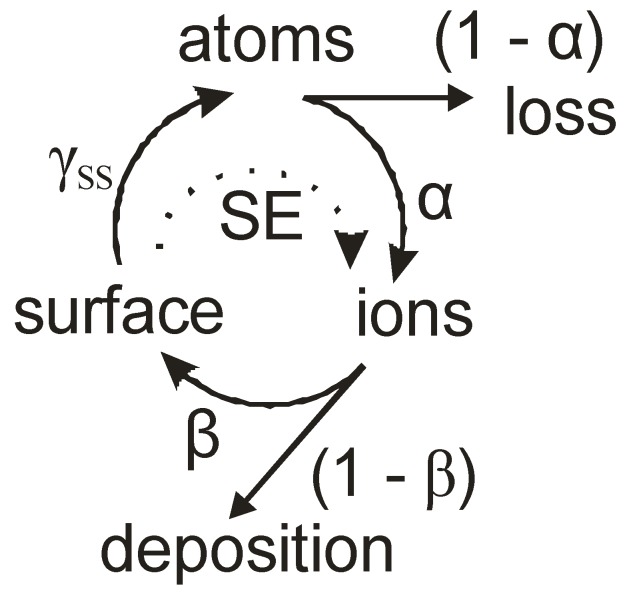
Schematic presentation of the self-sputtering process in high power impulse magnetron sputtering (HIPIMS), with loss terms and interactions with secondary electrons (SE).

The key parameter to describe this behavior is the self-sputtering parameter (see Figure 4 for a schematic representation) [56]

(1) $$\Pi =\alpha \beta \gamma_{SS}$$

where α is the ionization probability for a sputtered atom, β the probability for this ion to return to the sputter target and γ_SS_ the self-sputtering yield. For Π = 1, a sustained discharge without any additional gas is possible, for Π < 1 gas molecules, respective ions are necessary to maintain the sputter process, whereas at Π > 1 a runaway discharge only limited by the capacity of the power supply or the pulse length is encountered. Parameters α and β are always less than 1, thus γ_SS_ has to be larger than 1 to achieve unity for Π. For the self-sputtering yield, a strong dependency on the elemental composition is observed, with especially copper and silver exhibiting a very high self-sputter yield [23].

The ionization probability α is strongly enhanced by a high secondary electron yield as these electrons transfer energy from the electric field present in the cathode sheath towards the plasma. They are fed as hot electrons, replenishing energy loss in ionizing collisions, and maintaining the electron temperature [56]. Any deposition processes from this self-sputtering plasma in HIPIMS occur from the loss term associated with β. A maximum in the ion flux towards the substrate of (1–β) cannot be overcome. In reality, additional losses to other walls prevent the attainment of even this value. Consequently, the much higher ion bombardment from the deposited species in HIPIMS is obtained by compromising the deposition rate expected from conventional magnetron sputtering, leading to values typically of the order of 25–35% at identical average powers [57]. At the same time, a directed particle flux is emanating from the magnetron cathode, necessitating a mechanical rotation or translation system for non-planar substrates to be coated, as well as incurring complications during scaling for larger magnetron cathodes or substrates. Even though, no particulate problems have been observed for HIPIMS, in contrast to the following methods.

### 2.2. Vacuum Arc Deposition

In contrast, an ionization ratio of close to 100% is already achieved in vacuum arc deposition [29,58]. While a superficial similarity to the self-sputtering mode in HIPIMS is present, the underlying physical process and scaling lengths are completely different. A cathodic vacuum arc is characterized by plasma production at micrometer-size cathode spots which are rapidly moving across the cathode [59,60].

**Figure 5:**
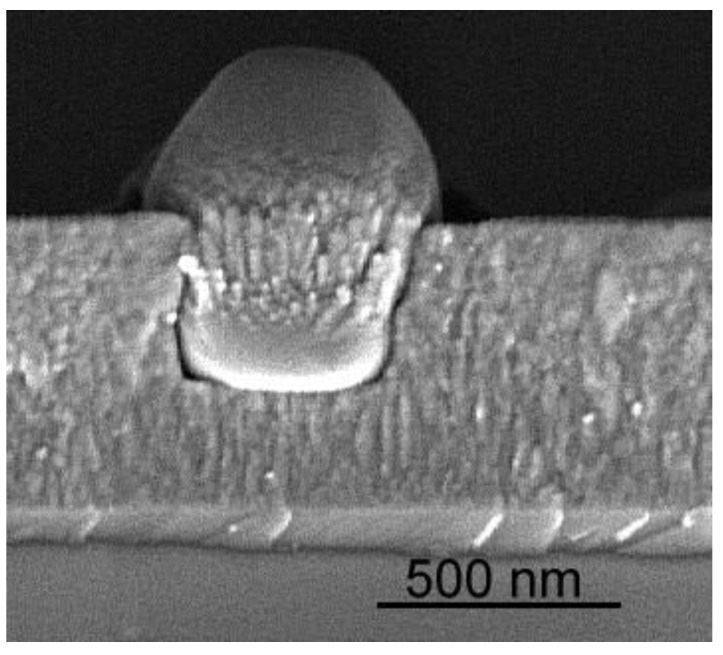
Cross section of TiO_2_ thin film with prominent macroparticle, subsequently partially coated at later stages of the deposition process.

A small, dense plasma cloud with plasma and power density of up to 10^26^ m^-3^ and 10^13^ Wm^-3^ respectively is generated by evaporation processes at the spot surface. Within a small space charge layer of 5–10 nm, a high electric field is accelerating ions towards the surface and emitting electrons—either by thermionic emission or field emission—towards the plasma ball, despite a rather low voltage drop of only about 50 V. Within the plasma ball, the electron beam is stopped by collisions and the vapor is heated and ionized, with thermal ionization dominating over direct impact ionization [29]. This central main plasma of the arc spot has a typical diameter of about 5–20 µm. Here, a local thermodynamic equilibrium is reached—albeit with the ion temperature lower than the electron temperature. Beyond this spot plasma, expansion of the plasma in the direction towards the anode is favored by the influence of the anode potential. While the ions are accelerated, the plasma density is rapidly decreasing, reducing the collision rate and freezing the plasma at a distance of less than 100 µm from the spot plasma. As a result, a supersonic ion flux with velocities of 1–2 × 10^4^ m/s and average charge states of 2^+^–3^+^, depending on the cathode material is observed [61]. It has to be noted that a parallel transport of ions and electrons towards the anode is occurring, with typically 10% of the total current as ions and 110% as electrons. A minimum current of about 50–75 A is necessary to allow these collective processes to occur, with an upper limit—in the pulsed mode—of several kA only due to the power supplies.

The plasma ball exerts a considerable pressure on the partially molten surface, reaching up to 40–50 bar and ejecting molten droplets, leading to macroparticles within the plasma stream [62]. As the presence of these macroparticles in deposited films, as shown in Figure 5, is a major obstacle for the broad application of cathodic arc deposition for high quality defect-free functional coatings, various filtering methods have been developed [63,64].

Nevertheless, a huge variety of applications is currently used in industry, foremost coatings for tools and decorative purposes, using planetary gear drives and multiple cathodes or a combination of magnetrons and arcs for homogeneity as well as multi-component coatings or direct multilayers [65,66]. Normally, elevated temperatures of at least 200–300 °C are employed to improve adhesion properties while additionally influencing the morphology and properties of the coatings themselves.

### 2.3. Pulsed Laser Ablation and Deposition

Pulsed laser ablation and deposition—or just pulsed laser deposition (PLD)—is a method similar to pulsed vacuum arc deposition. The depositing species are removed from a target material in short bursts resulting in a kinetic energy of these species of the order of 10–100 eV [31]. However, semiconducting or isolating materials can be used for PLD, which are not accessible for arc deposition. Normally, a congruent transfer of compound targets is observed [67], nevertheless multiple targets are commonly used as stoichiometry variations can be obtained in a much more easy and flexible way [68]. Similarly, elimination of particulates or droplets is still an ongoing research topic [69,70]. Currently, PLD is used widely for growth of high temperature superconducting oxides, dielectrics, ferroelectrics and semiconductors.

Developments in laser technology, especially short pulses, high power densities and reductions in price, all translated into a higher acceptance and utilization of PLD. Nevertheless, it is still restricted as the origin of the deposited material is a small and isolated area, while the production of functional coatings on large and complex shaped substrates is associated with sophisticated manipulation systems. However, the formation of coatings on inner walls in restricted geometries is possible [71]. Considerable effort was invested in reducing the available laser pulse length in PLD towards the femtosecond regime while maintaining the pulse energy. For such short pulses, no interaction of the laser beam with the ejected material is encountered, thus avoiding complicated secondary processes [72]. However, when reducing the pulse lengths to femtoseconds a change in the underlying physics of ablation and increased nanoparticle generation is observed, which can be an interesting goal itself while being more than a nuisance in thin film deposition.

### 2.4. Ion Beam Assisted Deposition

In ion beam assisted deposition, the arrival of low energy particles, e.g., from electron beam evaporators or effusion cells is complemented by high energy ions, typically in the range from 1 to 5 keV at current densities between 1 and 200 µA/cm^2^ [34]. A large amount of work concerning phase formation, heteroepitaxy, stress engineering and adhesion properties has been published and reviewed in the literature [33,73,74]. At the same time, technological applications including ferromagnetic thin film deposition or solar cells on temperature sensitive polymer substrates have been reported [46,75]. Advantageous is the independent variation of process parameters for ion beam and low energy particle flux, as the energy density can be varied independently from the particle flux density.

**Figure 6:**
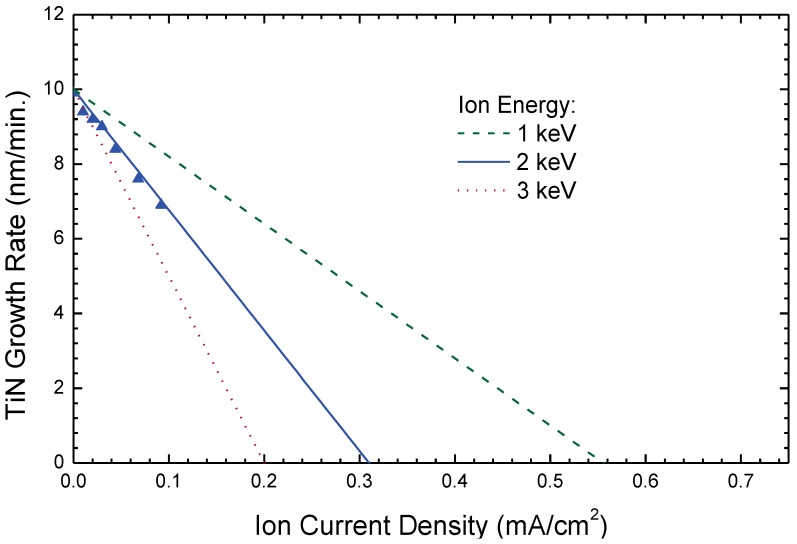
Variation of the growth rate for TiN thin films produced by ion beam assisted deposition (IBAD) with nitrogen ions of different energies at fixed titanium evaporation rate. As the sputter rate increases with ion energy in the range of interest, a higher ion energy leads to a reduced growth rate. The triangles indicate measured data points, the lines are from simulations using calculated sputter yields from Reference [79].

Disadvantages of the method include low growth rates compared to vacuum arc or magnetron sputtering due to low ion current densities even from modern ion sources, together with defect generation within the bombarded film, as low energy ions below 100 eV at sufficiently high current densities, and, finally, concurrent ion bombardment of large areas, are not feasible at the moment. At the same time, sputter removal of the growing film is a constant companion, setting limits for the upper ion current density, respective energy density, as shown in Figure 6.

The technological acceptance and utilization are closely connected with the development of modern broad beam ion sources [76,77], leading to recent developments of commercial dual ion beam systems for large area and flat substrates by different companies [75,78].

### 2.5. Plasma Immersion Ion Implantation and Deposition

The restrictions of a direct line of sight process can be overcome, at least partially, using a biased substrate. In PIIID, a condensable plasma—normally created by a cathodic arc—is combined with high voltage pulses of 1–10 kV applied directly to the substrate. Thus, a combination of low energy ion deposition with high energy ion implantation is obtained [36].

**Figure 7:**
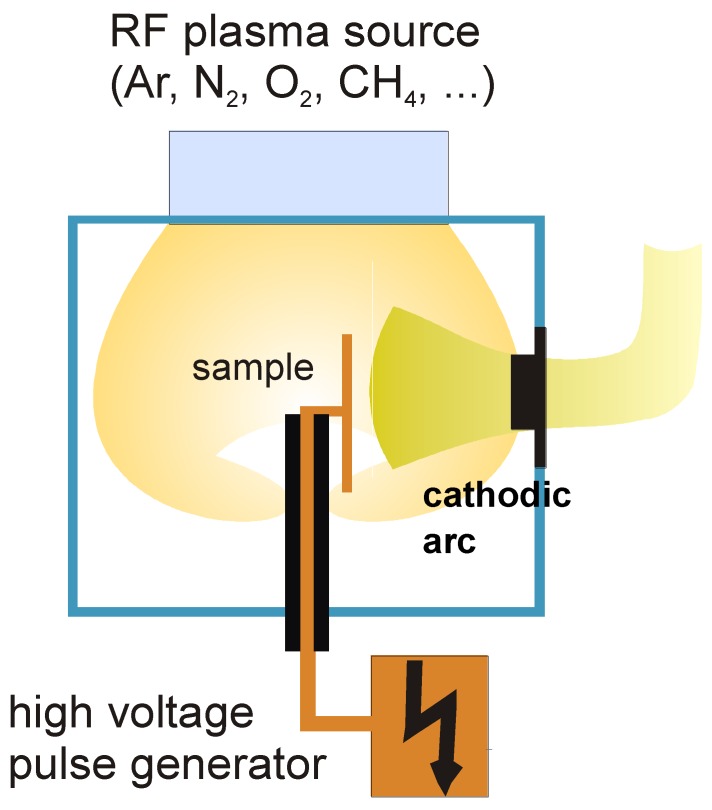
Presentation of a plasma immersion ion implantation and deposition (PIIID) system with filtered cathodic arc, auxiliary plasma source and high voltage pulse generator.

However, for non-planar substrates a complex situation is encountered, as shown in Figure 7. The flow of the condensable metal plasma can be described by magnetohydrodynamics, similar to a fluid moving around an obstacle in fluid mechanics. However, when encountering a surface, the metal ions will stick to it. Without high voltage pulses, the plasma sheath is very small, less than 100 µm, thus the local deposition rate closely follows the plasma flow. In contrast, its width can reach between 1 and 10 cm during the pulses, depending on the orientation in the plasma stream.

On the upstream side, no plasma presheath is present as the Bohm criterion is over-fulfilled [79], while it is re-established on the downstream side where the ions move away from the substrate, with a drop in the plasma density by a factor of 5–10 [80]. As a result of this, a very strong decrease of the layer thickness on areas not exposed to the plasma stream is observed [81]. A further complication is the existence of high energy ions producing defects deep in the bulk which will not be annealed for lower temperature deposition processes [82]. While a large number of groups, worldwide, are experimenting on laboratory scale, no commercialization of this processing method is known.

## 3. Thin Film Properties

All the thin film properties presented in this section depend on the provision of additional energy, preferably kinetic energy or potential energy from multiple charged cluster ions. However, additional factors such as chemical affinity, cohesive energy, orientation dependent surface energy and potential barriers encountered in volume, grain boundary or surface diffusion processes, always play a very important role. Hence, the mobility, respective ion damage, has to be seen in this light.

Radiation damage is the natural result of ion bombardment [25], which can be partitioned in nuclear and electronic energy loss. The former process describes momentum and energy transfer from the implanted particle towards nuclei of substrate atoms, while the latter is used to depict inelastic interactions with the electron system, leading to excitations on a timescale of less than 100 fs. In the energy range of 0.1–10 keV, the elastic nuclear energy loss dominates as long as projectile atoms with a mass equal or larger than carbon are used. For longer times, the excited volume increases from a few atoms towards several thousands of atoms while the average energy or—more precise the temperature of this region—translates to a few thousand or, at later stages, of several hundred K, returning to thermal equilibrium within less than a millisecond.

A multitude of parameters is experimentally (and theoretically) accessible, including the atomic structure of the interface to the substrate, important for electronic defects and adhesion, morphology and phase formation, which have to be looked upon separately. As long as no single crystalline films or amorphous films are obtained, the texture, *i.e.*, the distribution of crystallographic orientations of a polycrystalline film, can be influenced by the ion bombardment during deposition, as well as the intrinsic mechanical stress.

### 3.1. Interface Mixing

The process initially active during the energetic deposition of functional coatings is ballistic ion mixing leading to atomic relocation of the substrate material induced by the energy loss during the stopping of incident ions [83]. Besides an increased roughness of initial substrate surface, intermixing of the substrate and the grown film can occur, leading to an increased adhesion of the film on the substrate. An auxiliary process with a similar origin is surface sputtering, where the energy and momentum transferred to substrate atoms is sufficient for the secondary particle to be removed from the surface [84]. However, additional, chemical effects will arise from the respective mixing energy, with miscible systems showing broader interfaces than immiscible systems [85]. When depositing electronically active components, interface mixing has to be avoided as additional, unwanted interface states will arise. Next to reducing the maximum particle energy, which reduces the transient particle energy within the collisions, chemical mixing effects have to be circumvented in these cases.

**Figure 8:**
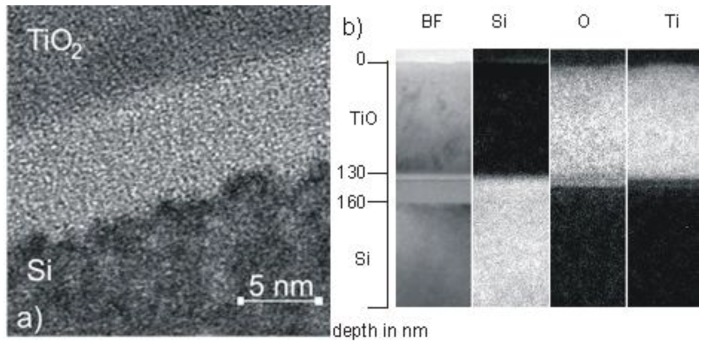
TEM image of the interface of a TiO_2_ sample deposited with (a) 1 kV voltage pulses on Si(100) and (b) 10 kV bias voltage [86].

An example is shown in Figure 8, where TiO_2_ thin films deposition onto crystalline Si substrate is presented using PIIID at different pulse voltages and constant duty cycle of 9% (pulse length 30 µs, repetition rate 3 kHz) [86]. With 1 kV pulse bias, a thin, amorphized interlayer with a thickness of about 5 nm and a corrugated interface towards the Si substrate is observed in the high resolution viewgraph in Figure 8.a. In contrast, thin films deposited with VAD without additional bias—corresponding to an average ion energy of around 50–75 eV—show an even thinner, but still noticeable interface [86]. When the pulse voltage is further increased to 10 kV, the thickness of the amorphous zone increases to 30 nm (as depicted in the BFTEM picture in Figure 8b). The growth rate of a little bit more than 2 nm/s has to be compared to projected ion ranges of 5 and 25 nm for low and high voltages, respectively, as calculated by SRIM for titanium ions [84]. The ballistic penetration depth of oxygen ions into silicon is nearly twice as large as that of titanium ions. However, beyond the purely ballistic effects, the width of the intermixed zone—and hence the effectiveness of the adhesion enhancement—is mainly determined by the mixing efficiency rather than the ion range [87]. In the system Ti-O-Si, a chemically driven effect can be assumed to dominate the ion mixing, similar to large amount systems investigated so far.

### 3.2. Morphology

The morphology of the growing film, tending towards polycrystalline at lower temperatures and faster deposition rates under consideration here, can vary significantly, depending strongly on substrate temperature, ion current and ion energy. A convenient way to depict general tendencies is a structure zone diagram [22]. The idea of such a diagram came originally from Movchan and Demchishin [88] with modifications by Barna and Adamik [89] as well as Thornton [90] and Messier [91]. In the presented form by Anders [22], it is extended and now proposed to cover any energetic deposition from cathodic arc deposition, HIPIMS, and other forms of PVD.

**Figure 9:**
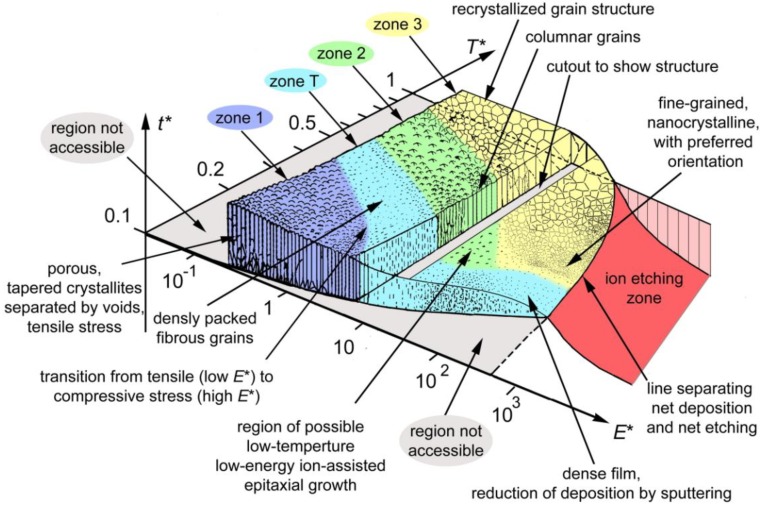
Structure zone diagram applicable to energetic deposition as a function of the generalized temperature T* and the normalized energy flux E*; t* represents the net thickness. The boundaries between zones are gradual and for illustration only. Reprinted from [22], Copyright 2010, with permission from Elsevier.

While many primary parameters such as target current, voltage, pressure or substrate distance will affect growth processes, they are impractical for comparison or for fundamental analysis. Hence—even for a qualitative presentation as in Figure 9—the parameters directly controlling the film growth process must be displayed. Thus, a generalized temperature T* (on a logarithmic scale) including the substrate temperature and any shift caused by the potential energy of particles arriving on the surface, together with a normalized energy E* (also on a logarithmic scale) describing displacement and heating by the kinetic energy of bombarding particles, is used. Additionally, an effective thickness including consequences of densification and sputtering, even up to dominating etching effects, is presented on the third axis.

With increasing substrate temperature, a transition from a porous phase (zone 1), towards densely packed fibrous grains (zone T), followed by columnar grains (zone 2) and finally a recrystallized grain structure (zone 3) or a zone of single crystals, is observed. As a result of ion bombardment, thermal energy is replaced by kinetic energy, allowing a similar morphology at different combinations of energy and temperature, leading to competing processes of defect generation, annealing, grain nucleation and crystallite growth. Thus the different zones 1, T, 2, and 3 are delimited by approximately diagonal lines. However, as mentioned previously in Section 2.4, an excessive ion bombardment leads to a domination of sputtering processes, thus reducing the effective, remaining film thickness. Additionally, texture effects and nanocrystalline grains may be encountered at high ion fluences. However, additional phase formation processes, especially of compounds with widely differing melting temperatures may complicate the picture in reality

### 3.3. Texture

Especially for columnar growth modes assisted by energetic particles, the evolution of the film is always associated with a preferred orientation or crystalline texture and subsequent development of this texture during progressive growth [92]. There is a strong correlation between the texture evolution and the process parameters such as ion energy, ion current density (ion/atom arrival ratio), film thickness, incident ion angle and substrate temperature. Different models exist to explain the preferred orientation in the film growth, where the channeling direction [42,93], preferential sputtering [38,94] or deformation energy minimization [41,95] are proposed to determine the final texture.

The Ti-N system has often been used to test the film growth models. An alteration of the preferred orientation from (200) and (111) to (220) orientation was found with increasing the average particle energy [96,97] in ion beam assisted deposition. Correspondingly, an influence of the texture on the mechanical properties is observed, with (200) oriented films showing a higher hardness and better wear resistance compared to films with a {111} texture [98,99].

**Figure 10:**
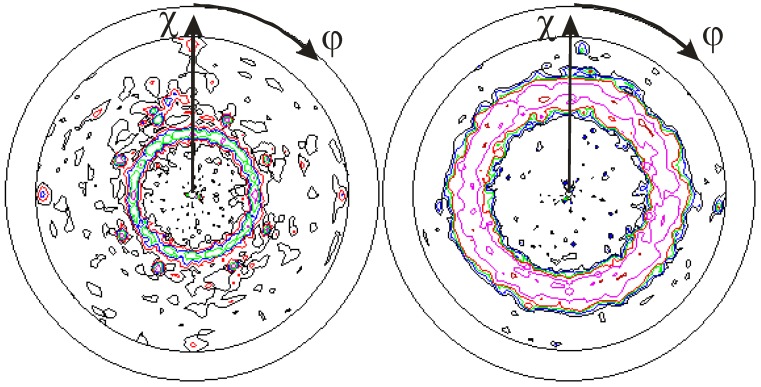
Depiction of TiN {110} and {200} fiber texture, as obtained from {111} pole figures, for plasma immersion ion implantation and deposition (PIIID) at different pulse voltages of (a) 1 kV and (b) 10 kV at fixed duty cycle of 9% [100].

**Figure 11:**
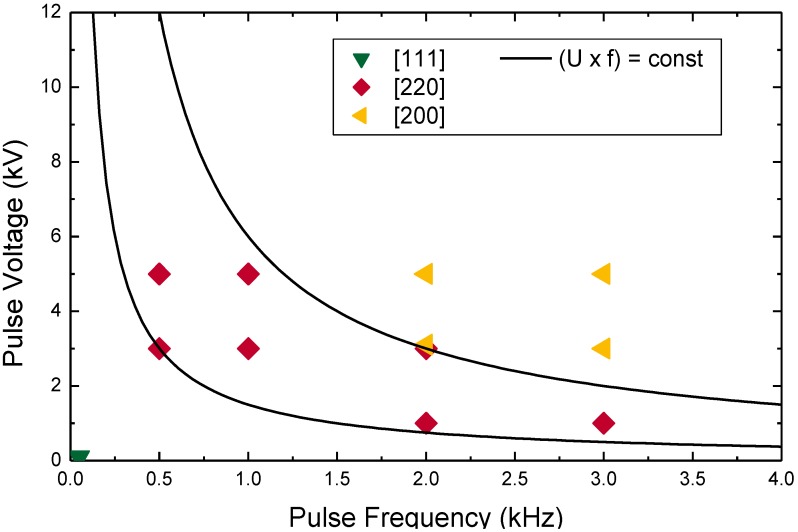
Observed texture evolution as a function of pulse voltage and frequency for the formation of TiN by PIIID [101]. The data points show the measured texture for thin film produced at different voltage/frequency combinations, the thin lines show the approximate texture transitions boundaries when assuming that the average energy alone is the dominating factor to determine the texture.

Using PIIID, a controlled variation of the incident energy flux is possible. A {200} fiber texture develops for sufficiently high-ion energies, 3 kV pulses or higher in the presented experiment (at 9% duty cycle, repetition rate 3 kHz, pulse length 30 µs), with the fiber axis parallel to the ion incident angle, *i.e.*, normal to the surface [100]. This fiber texture can be identified from the ring structure seen in Figure 10b centered at a polar angle χ = 55°. A {110} texture was measured for a lower-pulse voltage of 1 kV, indicated by the smaller ring centered at χ = 35° (see Figure 10a). Expanding the measurements to different pulse voltages and pulse lengths, *i.e.*, a varying energy flux density towards the substrate [98], a good agreement between the product of voltage and frequency with the observed texture is obtained [101], as shown in Figure 11. Hence, variation of current density or ion eney is producing the same effect. Similar diagrams can be found for carbon and AlN in the literature [102].

**Figure 12:**
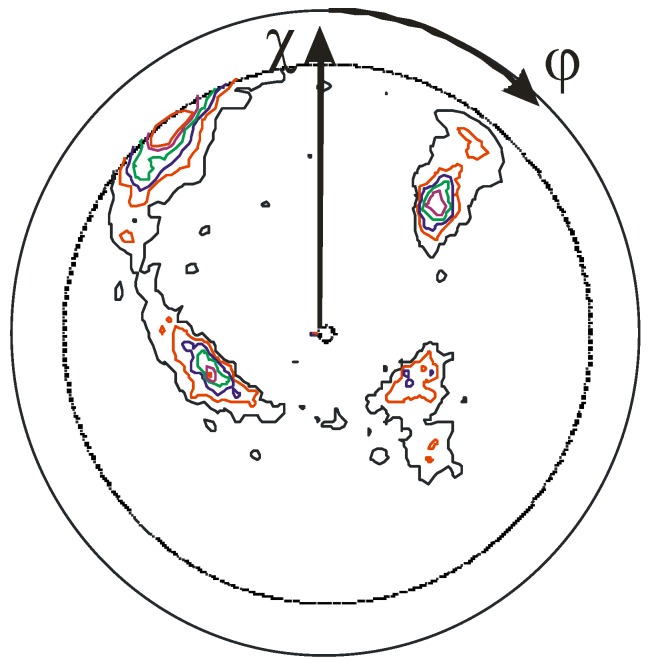
{111} TiN pole figure indicating a medium to weak biaxial texture with the (200) orientation tilted by about 20–30° from the surface normal, as seen in the shift of the center of the ring-like structure towards χ = 20–30° and the formation of four, more or less pronounced pole density maxima inside the ring structure, in contrast to Figure 10b.

The observations discussed in this subsection so far are only valid for normal incidence, thus orientation effects of the substrate normal and the ion beam incidence angle act in parallel. When changing the ion beam direction to selected crystallographic directions, the establishment of a biaxial alignment of the functional thin films is possible, as shown—again—for the system Ti-N with ion beam assisted deposition [103]. When comparing the texture evolution for off-normal ion bombardment for TiN, VN and CrN under identical conditions, additional influences of sputter yields and radiation damage on the orientation distribution are observed [104].

Using ion beam assisted deposition homogenous coating of large areas is possible with the ion beam arriving at the same angle at every position. In contrast, for vacuum arc deposition and PIIID, current density effects and variations in the angle of incidence are observed when moving from the center towards the edge. Thus, a continuous tilt of the texture and the columns constituting the film in the direction of the incident ion flux as a function of position on the sample is observed [105]. Furthermore, while reducing the symmetry of the ion flux, e.g., by tilting the sample relative to the incident ion beam, selected positions near the substrate edge show a transition from a fiber texture towards a biaxial texture, as depicted in Figure 12. Besides using the morphology of the growing film to elucidate the local ion flux as a function of the position on the substrate, an adjustment of texture on substrates according to the local loading conditions is a promising option in applications, as the texture is correlated with mechanical and tribological properties as well.

### 3.4. Mechanical Stress

**Figure 13:**
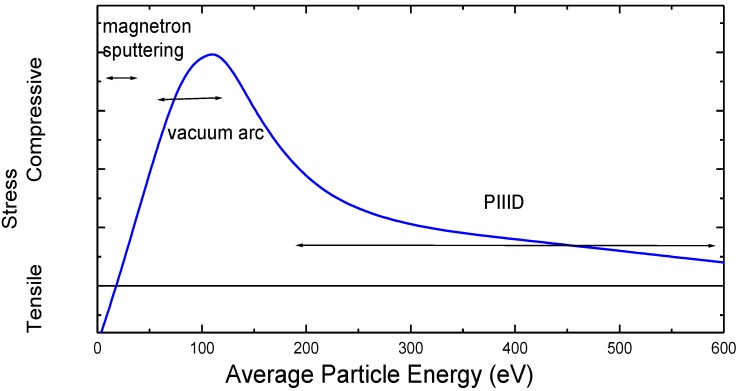
Evolution of intrinsic stress as a function of deposited energy. Typical energy regions for different processes are indicated.

In addition to texture and morphology, the film stress is determined by the deposited energy (as shown in Figure 13). At low energies, the films are found to be porous with a large void fraction and tensile intrinsic stress [106]. With increasing the average energy per particle to a few tens of eV, there is a transition to compressive stress as the material is densified by the impinging ions. Increasing the deposited energy even further, a maximum value for the stress is observed, ideal for the formation of cubic BN [107] and diamond-like carbon (DLC) coatings with a high sp^3^/sp^2^ ratio [108]. Beyond that value, stress relaxation by thermal spikes is proposed [109], accompanied by a slight reduction in density, which can be observed in several different materials, e.g., t-a:C or AlN [110,111].

While a continuous adjustment of the average particle energy is possible in IBAD processes, e.g., by changing the current density, the higher kinetic energy and charge-states of ions employed in vacuum arc deposition leads to a rather high threshold in energy [112]. Thus, when using PIIID with high voltage pulses in addition to the plasma stream from an arc, a stress value close to the maximum is observed even at low, additional ion bombardment during the pulses. By increasing the pulse frequency and voltage, a direct stress relaxation is possible. At a voltage of 5 kV, a duty cycle of 5% is sufficient for a reduction by more than 90%. Thus stress engineering can be used to obtain thick, well-adhering and relaxed c-BN or hard DLC coatings [113,114].

### 3.5. Phase Formation

For pure ion implantation at elevated ion energies beyond 10 keV, stable or metastable phase formation especially favoring high pressure, high temperature phases is often observed, as enough compressive stress is created by the forceful insertion of ions into the original atomic structure [25,105]. In contrast, during thin film deposition, and nucleation processes caused by ion bombardment are always accompanied by growth of grains or columns, making a direct distinction between both processes very difficult. From a practical point of view, this question seems to be rather irrelevant. However, when facing the scaling up of a process or a transfer to a different substrate where a different balance between substrate temperature and ion bombardment may be necessary, different technology adjustments are required to maintain the functionality of the coatings.

As several distinct processes may be influential for the phase formation, a clear separation of causal links, which is deemed necessary, yet is not always possible. When returning to DLC or BN, the major factor determining the sp^2^/sp^3^ ratio, respective the nucleation of cubic or hexagonal BN, the major factor is creation (or absence) of compressive stress and not the transient increase of the temperature during the stopping of the incident ions.

Detailed results for investigations of interchanging temperature and ion bombardment without additional stress effects are available, e.g., for the system Ti-O, which has been and is extensively investigated. Beside widespread use of TiO_2_ thin films in optics because of their high refractive index and their stability [115], TiO_2_ is known as one of the most effective photocatalysts with its photocatalytic behavior extensively studied [116,117]. In addition, photoinduced superhydrophilicity is reported for TiO_2_ with the water contact angle decreasing from 72° in the dark towards values of less than 10° or even 0° under illumination [118]. A large amount of work employs high deposition temperature of around 200 °C or more or post annealing-steps after magnetron sputtering [119,120], thus precluding the use of temperature sensitive substrates such a polymers.

**Figure 14:**
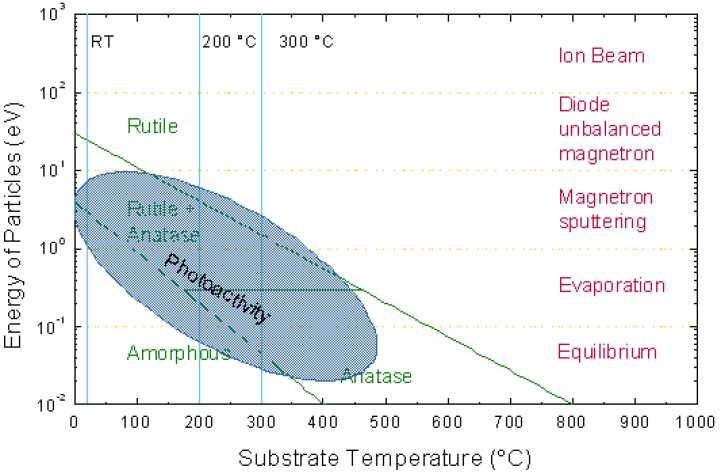
Identification of process window to obtain different phases as a function of substrate temperature and particle energy. Additionally, the region allowing photoactive thin films is indicated by the blue shaded region [124].

The phase formation itself shows a transition from amorphous via anatase at intermediate conditions towards rutile for high temperature, respective high particle energies [121], as schematically depicted in Figure 14. Temperature and ion energy seem to be interchangeable, however the plot apparently implies that an increase in the average particle energy by a factor of 10 leads to very similar results when increasing the temperature by 300 °C. This combination of logarithmic energy and linear temperature scale still necessitates further investigations. Similar results on the phase formation have been observed for pure implantation of oxygen into chemically pure titanium, where the high temperature-high pressure phase rutile was found to nucleate even at a temperature of 265 °C [122]. However, at temperatures between 250 and 600 °C, anatase is the fast growing phase whereas rutile is growing very slowly. Thus, depending on growth rate and deposition time, different anatase/rutile mixtures can be encountered. Furthermore, unequivocal phase identification by either XRD or Raman spectroscopy of microcrystalline or nanocrystalline components is rather difficult [123].

**Figure 15:**
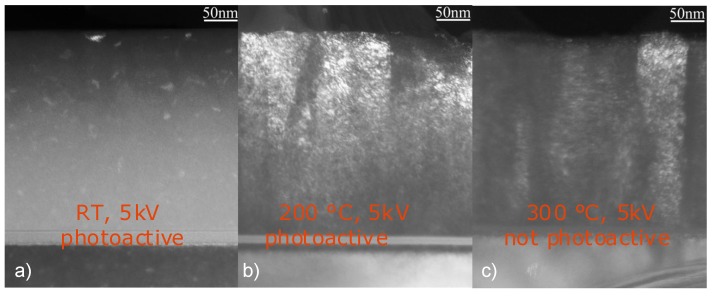
Transmission electron microscopy (TEM) dark field viewgraphs of three different samples, deposited at 5 kV pulse voltage at (a) room temperature, (b) 200 °C and (c) 300 °C.

However, a strong correlation of the phase composition—and not the morphology—with the photoactivity was observed, which can help in identifying the actual phase composition. Photoactivity was observed at room temperature and 200 °C, but not at 300 °C when applying 5 kV pulse voltages [124]. As can be seen in Figure 15, an amorphous phase with nanocrystalline rutile inclusions (indicated by the bright regions in the thin film in Figure 15a) was obtained at room temperature, while at higher substrate temperatures, columnar growth was observed.

## 4. Selected Applications

In this section, several recent examples for laboratory applications—some of them already transferred as a standard industrial process—are shown to elucidate the possibilities and limitations of thin film deposition employing energetic ions. The aim is to exemplify a few kinds of novel applications and possible further research topics helping to elucidate the full potential from them, in contrast to an exhaustive overview across the whole subject area. Semiconducting thin films, such used for blue or green LEDs or advanced coatings for tools employ energetic thin film deposition of course, but they are not within the scope of this review.

First the state of the art for advanced optical multilayers is shown, where the multitude of processing parameters is successfully adjusted to obtain an optimum interplay of different properties for the intended application. Here, the term “nanostructures” describes only the third dimension, the growth direction perpendicular to the substrate, while large areas of more than 0.05 m^2^ are nowadays coated homogeneously, respectively with intentional gradients across the substrate. Additionally, two types of nanostructured functional surfaces—chiral structures with real 3D sculpting and magnetic structures or dots—are presented. Here the main focus is presently on obtaining the nanostructures, either by top-down or bottom-up approach, and less on defining or even optimizing the “structural properties” as epitomized in the previous section.

### 4.1. Multilayers by Ion Beam Sputter Deposition

**Figure 16:**
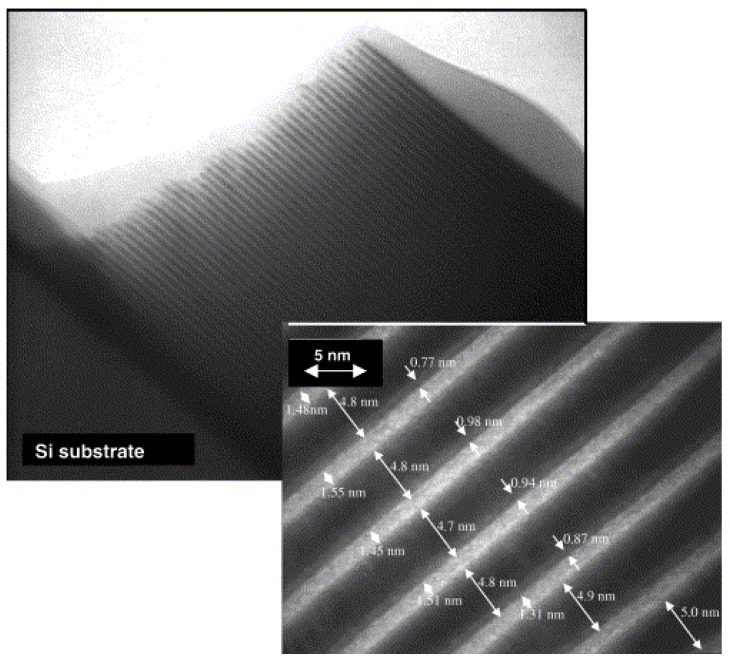
Cross-section TEM micrograph of a Mo/Si multilayer stack formed by dual ion beam assisted deposition. The inset shows in detail the thickness of the Si-layer (dark), the Mo-layer (bright) and the interface between Si and Mo. Reprinted from [34], Copyright 2002, with permission from Elsevier.

Mirrors and mask blanks for extreme ultraviolet lithography (EUVL) have been identified for semiconductor production at reduced pitch for more than a decade with early experiments starting before the end of the century [125]. Here mask blanks are more demanding than mirrors as a much lower defect density is permitted, in addition to the requirements of high homogeneity, low surface and interface roughness, low stress, high temperature and photon flux stability [126]. Next to pulsed laser deposition [127] and magnetron sputtering [128], ion beam sputter deposition has been substantiated to allow for improved layer smoothness, intermixing suppression and stress reduction, as shown in Figure 16 [129,130].

40–50 molybdenum/silicon bilayers of 3 and 4 nm thickness, respectively, are required for a maximum reflectivity of around 70% at a wavelength of 13.5 nm. One decisive parameter for the interface roughness, beside intermixing and interdiffusion, is the morphology of the single layers. Especially Mo is critical as a transition from an amorphous to a polycrystalline phase has been observed around a thickness of 2 nm [131]. Thus, the observed asymmetric Mo_x_Si_y_ interlayer thickness at Mo/Si and Si/Mo interfaces within the multilayers could be explained [132]. However, an influence of the deposition method was found with magnetron sputter deposition leading to a slightly earlier phase transition—around 2 nm instead of around 2.5 nm—compared to ion sputter deposition. Additionally, increasing the partial pressure of the sputter gas leads to an increase in the transition thickness [133]. Using real-time *in situ* spectroscopic ellipsometry, it is possible to investigate this phase transition in detail [134].

**Figure 17:**
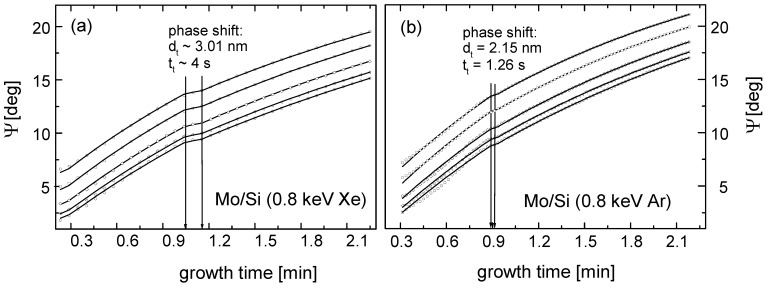
Real time spectroscopic *in situ* ellipsometry experimental (points) and model (solid lines) spectra of Mo layer growth in the (a) Xe sputter regime (E_ion_ = 0.8 keV, f_Xe_ = 1.6 sccm) and (b) Ar sputter regime (E_ion_ = 0.8 keV, f_Ar_ = 3.2 sccm). The different lines refer to different wavelengths [134].

The transition from amorphous to polycrystalline can be observed by the formation of a plateau in the otherwise continuously increasing spectroscopic angle Ψ, as shown in Figure 17. Interestingly, even for ion beam sputter deposition without background gas, the sputter ion species influence the morphology transition. With argon ions, exhibiting a higher average energy for particles backscattered from the target than xenon ions, an earlier transition, at a smaller thickness, is observed. While the metastable amorphous phase is stabilized by interface energies and elastic strain, a particle bombardment induced recrystallization due to an increase in the free energy by introducing strain is observed [134].

**Figure 18:**
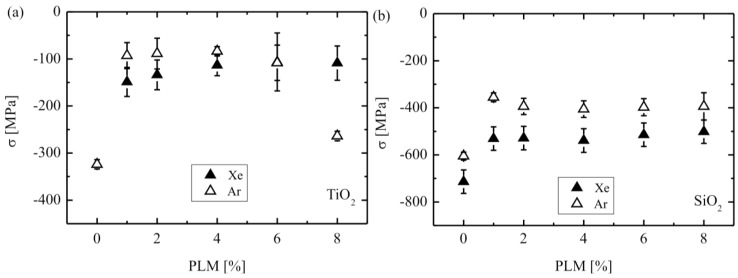
Film stress *versus* pulse length modulation of the assisting ion beam for **(a)** TiO_2_ and **(b)** SiO_2_ films grown with an ion energy of 1.2 keV of the assist ion beam. PLM indicates the pulse length modulation of the assist ion beam [135].

While in the previous example no additional assist ion source was employed to avoid excessive damage, defect creation and increased roughness, stress relaxation, respective tailoring for dielectric multilayers on very thin substrates necessitates dual ion beam deposition processes [135]. While aiming to provide highly reflective coatings for advanced laser applications, including micro mirrors in laser scanners or endoscopes, on very thin substrates of less than 50 µm thickness, it was observed (see Figure 18) that the compressive stress of TiO_2_ and SiO_2_ layers could be considerably reduced by employing a second, assisting ion source directed towards the substrate. By increasing the ion flux, respective the pulse length modulation, a stress reduction by 25–65% could be obtained, additionally depending on the ion species and the material system.

The underlying processes defining the optical as well as mechanical properties, including substrate adhesion as thermal cycling is prominent for these kinds of optical mirrors, are all well understood. Parameter optimization and transfer from laboratory towards industry, as well as upscaling have all been addressed within the last decade, thus allowing successful applications and commercialization.

### 4.2. Chiral Nanostructures by Glancing Angle Deposition

**Figure 19:**
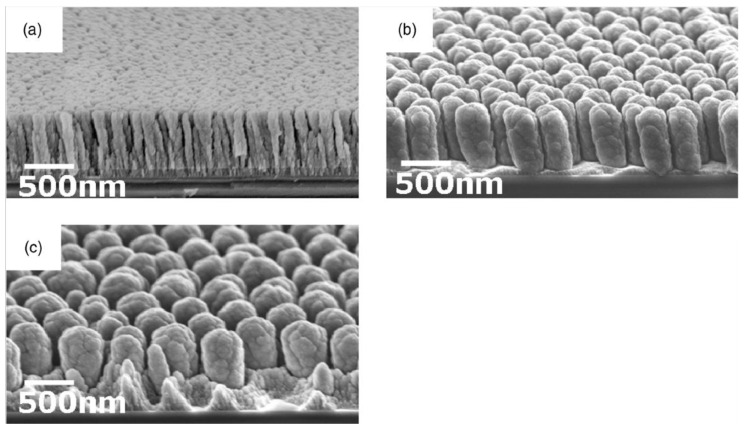
15°-tilted cross-sectional SEM micrographs of Si nanocolumns: (a) without template pattern, (b) honeycomb pattern, and (c) hcp pattern. Reprinted with permission from [151]. Copyright 2008, American Institute of Physics.

As ion bombardment of growing thin films inevitably leads to a surface morphology characterized by a reduced roughness due to the enhanced surface mobility (compare to Figure 2), more sophisticated approaches are necessary to obtain sculptured, three-dimensional structures (as shown in Figure 19), which entail a variety of applications. These include three-dimensional photonic band gap materials [136,137], thermal barrier coatings [138], optical filters [139], sensors or magnetic storage devices [140,141]. Here, glancing angle deposition (GLAD) where the incident particle flux is arriving at an extreme grazing angle of about 85° from the surface normal [142,143], together with substrate rotation, is gaining more and more attention with the particle flux provided by evaporation [144], magnetron sputtering [145] or ion beam sputtering [146].

In particular, deposition parameters (rotation frequency, substrate temperature, deposition angle) influence the nanostructure morphology, especially the internal structure presently less thoroughly investigated, and area density. In the course of deposition a structure broadening with a critical height was observed at which neighboring nanostructures merge. At elevated substrate temperatures (300–360 °C) the nanostructure formation is determined by adatom diffusion [147,148]. Furthermore, the GLAD deposition of ordered nanostructures can be achieved by substrate preparation via nanosphere lithography [149]. Here, honeycomb and hexagonal patterns of extremely uniform Si nanostructures of different shape can be produced. By varying the tilt angle in the range from 65 to 88°, the formation of sculptured thin films is possible [150]. Thus, nanostructures of small aspect ratios can be used for the fabrication of sub-wavelength-antireflection coatings in the deep ultraviolet spectral region.

A recent variation of GLAD or oblique angle deposition (OAD) techniques, called conformal-evaporated-film-by-rotation (CEFR), has demonstrated its potential in the fabrication of high-fidelity replicas of biological templates [152] and micromechanical systems (MEMS) [153]. Here, the combination of PVD and substrate tilting and rotation leads to the conformal coating of planar as well as curved surfaces. However, coating of complex 3D objects is not possible with GLAD, in addition to very low deposition rates encountered in these techniques. Moreover, an understanding of the underlying processes and the actual tailoring of the nanostructure properties beyond the arrangement itself—which nevertheless are crucial in defining the optical properties—is just the beginning. Furthermore, the presently employed materials are mostly Si, SiO_2_ or Ge, which is a rather small and limited subset of materials currently employed in materials science and, especially, for biological applications not the most favored group of materials. There is still a large unfilled demand for advanced nanostructured implants or templates with a defined topography and chemical activity [154,155].

### 4.3. Magnetic Nanostructures

In addition to optical nanostructures, magnetic nanostructures present a vast area of current and potential applications, e.g., for sensors, patterned media or novel magnetic properties [156,157]. Here, ion implantation is able to allow local tailoring of magnetic properties [158] in contrast to lithographic control of film growth or etching [159]. In specific substrates, even sub-100 nm patterning by focused ion beam (FIB) is possible, creating ferromagnetic patterned structures within a paramagnetic matrix [160]. However, a top-down approach is used in either method.

**Figure 20:**
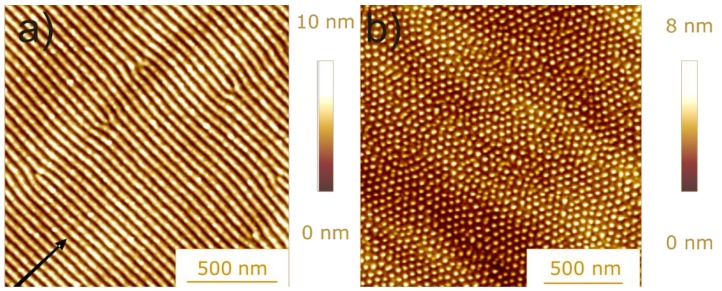
Surface topography after ion bombardment: (a) Si surface, 1200 eV Ar^+^, 15° off-normal; the arrow indicates the projected ion beam direction. (b) Ge surface, 2000 eV Xe^+^, 20° off-normal [164].

In contrast, removal of surface atoms by sputtering, together with radiation damage and the insertion of the foreign atom into the host material, leads in general to a modification of the surface topography, as shown in Figure 20. For semiconductors, nanostructures including regular ripples [161] or dot arrays [162] can be observed. These former structures are explained by a competition of sputtering and surface relaxation mechanisms [163], with typical structure periods in the range between 10 and 100 nm, albeit only with aspect ratios below one [161]. Here, self-organization processes allow a bottom-up approach towards surface patterning without any masks or focused particle beams, thus reducing costs as well as processing times.

However, the challenge is to employ this technique for fabrication of nanomagnet arrays. One possibility is to use a nanostructured template for thin film deposition. Shadow deposition at moderate angles between 20 and 45° allows the formation of nanomagnet sizes below 20 nm and storage densities of at least 0.2 Tbit in^-2^. [165]. Alternatively, deposition of homogeneous thin films, covered by an overlayer susceptible to pattern formation and subsequent ion erosion of this top-layer, leading to a structured surface topography with local variations in the effective sputter yield, will lead to ordered arrays of magnetic nanoparticles [166,167]. While a variety of materials is introduced here, owing to the necessity of employing, respective obtaining magnetic materials, the limitations of untried—or currently still neglected—attention to the physical fundamentals within the modified surfaces and upscaling, as already mentioned in the last subsection, apply here, too. Nevertheless, this combination of 3D-sculpting and surface functionalization on the nanoscale presents a very promising research topic.

## 5. Conclusions

For functional coatings, with optical, magnetic or otherwise applications, two important effects have to be addressed during deposition. First, a trade-off between enhanced surface mobility and defect generation has to be achieved with the creation of increased interface roughness and electronic defect density *vs.* stress relaxation and texture formation. A specific balance has to be found for each application. Secondly, top-down approaches for formation of nanostructures contrast with bottom-up methods. Nanospheres are nowadays employed as masks for nanostructures while the understanding and utilization of self-organization phenomena is still improving, respective expanding. Nevertheless, a large portfolio of deposition methods is available, allowing for the formation of sophisticated nanostructures as long as the underlying processes are understood.

Physical vapor deposition for formation of advanced functional coatings or nanostructured surfaces is a sizable field of technologies as no single method is dominating the field, neither in the laboratory nor in industrial applications. While fast deposition processes and the ability to coat complex shaped 3D substrates are desirable for low-cost, high-volume applications, thin films without defects and the ability to form nanostructures are the domain for technologically advanced coatings where energetic particles are employed for a multitude of adjustments, including morphology, texture and stress. For this class of applications IBAD processes are dominating for large and planar—or at least weakly curved substrates—with HIPIMS starting to create a huge amount of interest and research, due to a better control of the particle energy and less macroparticles. Whether it can supersede vacuum arc deposition, PIIIID or magnetron sputtering will be decided within the next years. The main problem for HIPIMS nowadays are low deposition rates and—compared to dual ion beam sputter deposition—no efficient way to control the particle, momentum and energy flux independently. Yet, here the verdict is still open with strong ongoing discussion and research efforts within the scientific community.

While a large amount of literature exists on the different phenomena and resulting thin film properties during deposition processes, the multitude of accessible effects leads to a large variability between different materials systems. Thus, the transferability is restricted to subsets of similar chemical reactivity, view for instance the transition metal nitrides and carbides often employed for wear resistant tool coatings. However, no fundamentally new physics can be expected from energetic thin film deposition processes.

The current trend towards real three-dimensional nanomaterials, in distinction from nanolayers where the film thickness is very much smaller than the lateral dimensions, is still gaining momentum. Currently, several interesting and novel ways are being explored to obtain these kinds of structures, albeit with no clear favorite at the present. Yet, while the “macrostructure” formation is eager investigated, the secondary properties—such as phase formation, chemical segregation or stress relaxation—are not featuring prominently. Partially, this is certainly due to the relative infancy of some methods where the focus is primarily on understanding the deposition and only secondarily on optimizing the properties. However, it has to be mentioned that even the most advanced analytical techniques are running into problems: both from the very small volume available for analysis and for statistical comparisons between different, isolated nanostructures. Even so, the present research has scratched only on the surface of the vast possibilities opening up in the old, but still ever evolving field of energetic thin film deposition for functional, nanostructured coatings.
